# Distribution of *CYP2D6* Alleles and Phenotypes in the Brazilian Population

**DOI:** 10.1371/journal.pone.0110691

**Published:** 2014-10-20

**Authors:** Deise C. Friedrich, Júlia P. Genro, Vinicius A. Sortica, Guilherme Suarez-Kurtz, Maria Elizabete de Moraes, Sergio D. J. Pena, Ândrea K. Ribeiro dos Santos, Marco A. Romano-Silva, Mara H. Hutz

**Affiliations:** 1 Departamento de Genética, Universidade Federal do Rio Grande do Sul, Porto Alegre, Rio Grande do Sul, Brazil; 2 Programa de Farmacologia, Instituto Nacional de Cancer, Rio de Janeiro, Rio de Janeiro, Brazil; 3 Departamento de Fisiologia e Farmacologia, Universidade Federal do Ceará, Fortaleza, Ceará, Brazil; 4 Departamento de Bioquímica e Imunologia, Universidade Federal de Minas Gerais, Belo Horizonte, Minas Gerais, Brazil; 5 Laboratório de Genética Humana e Medica, Universidade Federal do Pará, Belém, Pará, Brazil; 6 Instituto Nacional de Ciência e Tecnologia de Medicina Molecular, Universidade Federal de Minas Gerais, Belo Horizonte, Minas Gerais, Brazil; Centro de Pesquisa Rene Rachou/Fundação Oswaldo Cruz (Fiocruz-Minas), Brazil

## Abstract

The CYP2D6 enzyme is one of the most important members of the cytochrome P450 superfamily. This enzyme metabolizes approximately 25% of currently prescribed medications. The *CYP2D6* gene presents a high allele heterogeneity that determines great inter-individual variation. The aim of this study was to evaluate the variability of *CYP2D6* alleles, genotypes and predicted phenotypes in Brazilians. Eleven single nucleotide polymorphisms and *CYP2D6* duplications/multiplications were genotyped by TaqMan assays in 1020 individuals from North, Northeast, South, and Southeast Brazil. Eighteen *CYP2D6* alleles were identified in the Brazilian population. The CYP2D6*1 and CYP2D6*2 alleles were the most frequent and widely distributed in different geographical regions of Brazil. The highest number of *CYPD6* alleles observed was six and the frequency of individuals with more than two copies ranged from 6.3% (in Southern Brazil) to 10.2% (Northern Brazil). The analysis of molecular variance showed that *CYP2D6* is homogeneously distributed across different Brazilian regions and most of the differences can be attributed to inter-individual differences. The most frequent predicted metabolic status was EM (83.5%). Overall 2.5% and 3.7% of Brazilians were PMs and UMs respectively. Genomic ancestry proportions differ only in the prevalence of intermediate metabolizers. The IM predicted phenotype is associated with a higher proportion of African ancestry and a lower proportion of European ancestry in Brazilians. PM and UM classes did not vary among regions and/or ancestry proportions therefore unique *CYP2D6* testing guidelines for Brazilians are possible and could potentially avoid ineffective or adverse events outcomes due to drug prescriptions.

## Introduction

Cytochrome P450 (CYP) is a superfamily of proteins responsible for metabolizing a wide spectrum of different substrates, both endogenous and xenobiotics. The CYP system has a central role in pharmacogenetics area since most medication prescribed is substrate for these enzymes. CYP2D6 enzyme is one of the most important members of this family. Though CYP2D6 comprises only 2% of human hepatic CYP450 enzymes, it metabolizes more than 25% of all currently prescribed medications and it is not induced by environmental factors [1]–[6].

*CYP2D6* spans a 4.3 Kb region on chromosome 22q13.1 and forms a cluster with two pseudo-genes *CYP2D8P* and *CYP2D7P* [7]. At present, more than 100 allelic variants and sub variants are described [8]. They are the result of different mutation types mainly single nucleotide polymorphism (SNPs), but insertion/deletion (indel), gene rearrangements, and deletion or duplications of the entire gene have also been observed. This high polymorphic level results in four CYP2D6 phenotypes: poor metabolizers (PM; two inactive alleles), intermediate metabolizers (IM; one reduced activity allele and one inactive allele or two reduced activity alleles), extensive metabolizers (EM; at least one functional allele), and ultrarapid metabolizers (UM; three or more functional copies) [9]–[11].

*CYP2D6* allele and phenotype frequencies have been investigated in all major ethnic and geographical populations and present significant differences in allele frequencies within and between populations [2],[10],[12]–[14].

Brazilians form one of the most heterogeneous populations in the world. Amerindian people occupied the Brazilian territory when the Portuguese arrived in 1500 and colonized the country. Then between the 16^th^ and 19^th^ centuries, Africans were brought to Brazil as slaves. In addition to the Portuguese, other migratory waves occurred in the 19^th^ and 20^th^ centuries, mainly from Italy, Germany and Spain [15]. All of these migratory events contributed to the formation of a multi-ethnic and highly admixed population. This heterogeneity was documented in several genetic studies that used uniparental or autosomal markers to demonstrate a typical, although non-uniform, tri-ethnic (European, African and Amerindian) pattern for the Brazilian population. This admixture process occurred in different ways in the various geographic regions of the country. In Northeastern Brazil, the African contribution is high; in the North, the contribution of Native Americans is pronounced; and in the South, the influences are predominantly European with reduced Amerindian and African contributions when compared to other Brazilian geographic regions [16]–[20].

Since the knowledge of allele distribution and frequency is required to effectively translate pharmacogenetics to the clinics and given the paucity of *CYP2D6* data in the Brazilian population, the purpose of this study was to evaluate the influence of ethnic classifications, geographical origins and genetic ancestry in *CYP2D6* alleles, genotype and predicted phenotype distributions in Brazilians.

## Results

A total of 18 different alleles and duplications were identified (Table 1). CYP2D6 *1, *2, *4, *5, *10, *17, *29, *35, *41, *1×2, *2×2 alleles were polymorphic in all Brazilian regions. These 11 alleles account for more than 97% of all investigated chromosomes. Some alleles and/or duplications were found exclusively in one region, e.g. CYP2D6*34 and CYP2D6*35×2 in the Northeast, CYP2D6*17×2 in the South (Table 1). The genotypes observed in Brazilians are listed in Table S1. *CYP2D6* copy number varied from three to six (Table 2). The frequency of individuals with more than two copies ranged from 6.3% (in Southern Brazil) to 10.2% (Northern Brazil). According to the genotyping procedure, the duplications could not be unambiguously assigned to an allele in some heterozygous individuals, therefore an underestimation of some duplicated alleles occurred. Overall *CYP2D6* allele distribution is homogeneous in Brazil. AMOVA showed that more than 99% of the variability observed in allele frequencies is due to differences among individuals; the differences between regions and self-reported skin color accounted respectively for 0.2% and 0.5% of the total variance observed (Table 3). Pairwise F_ST_ results obtained among regions and among self-reported skin color are shown in Table 4. All F_ST_ estimates were low and no differences among regions or self-reported skin color were observed.

**Table 1:** *CYP2D6* allele frequencies in Brazil.

Allele frequency % (Number of chromosomes)					
*CYP2D6* allele	Total sample	North	Northeast	Southeast	South
*1	39.9 (814)	40.3 (198)	36.8 (192)	40.7 (210)	41.9 (214)
*2	21.5 (438)	24.4 (120)	24.1 (126)	18.8 (97)	18.6 (95)
*3	0.7 (14)	0.6 (3)	0.8 (4)	0.6 (3)	0.8 (4)
*4	9.4 (191)	10.8 (53)	9.6 (50)	7.7 (40)	9.4 (48)
*5	4.6 (94)	2.7 (13)	5.4 (28)	5.4 (28)	4.9 (25)
*9	1.1 (23)	1.0 (5)	0.9 (5)	1.3 (7)	1.2 (6)
*10	2.05 (42)	1.4 (7)	1.7 (9)	3.1 (16)	1.9 (10)
*17	5.6 (114)	5.3 (26)	4.4 (23)	7.4 (38)	5.3 (27)
*29	3.4 (69)	2.2 (11)	2.3 (12)	4.3 (22)	4.7 (24)
*34	0.05 (1)	-	0.2 (1)	-	-
*35	2.6 (53)	2.2 (11)	3.1 (16)	2.9 (15)	2.2 (11)
*39	0.8 (17)	1.4 (7)	0.4 (2)	-	1.6 (8)
*41	5.5 (112)	4.1 (20)	7.8 (41)	5.0 (26)	4.9 (25)
*1×2	0.55 (12)	0.8 (4)	0.6 (3)	0.6 (3)	0.4 (2)
*1×3	0.05 (1)	0.2 (1)	-	-	-
*2×2	0.55 (11)	0.6 (3)	0.4 (2)	0.8 (4)	0.4 (2)
*2×5	0.05 (1)	-	0.2 (1)	-	
*4×2	0.2 (4)	0.4 (2)	-	0.4 (2)	-
*17×2	0.05 (1)	-	-	-	0.2 (1)
*35×2	0.05 (1)	-	0.2 (1)	-	-
Others^a^	1.3 (27)	1.6 (8)	1.1 (6)	1.0 (5)	1.6 (8)
Total number of chromosomes	2040	492	522	516	510

a SNP combination that could not be assigned to a known allele, for more information see Table S3.

χ^2^ = 70.184 and p-value = 0.069 for the comparison of the allele frequencies among the four regions.

**Table 2:** Copy number variation of the *CYP2D6* in the Brazilian population.

*CYP2D6* copy number	Total sample	North	Northeast	Southeast	South
3	67	21	18	14	14
4	16	3	4	7	2
5	3	1	2	0	0
6	1	0	1	0	0
Number of individuals with more than two copies	87 (8.5%)	25 (10.2%)	25 (9.6%)	21 (8.1%)	16 (6.3%)

**Table 3:** AMOVA results for the allele frequencies of the sample stratified by region and by self-reported skin color.

Source of variation	Percentage of variation	
	By region*	By skin color**
Among populations	0.2	0.5
Within populations	99.8	99.5
Fixation Index (Φ_ST_)	0.00164	0.0052
p-value	0.034	<0.00001

*Four Brazilian regions were considered: North, Northeast, Southeast, and South.

**Three skin color categories were considered: Black, Brown and White.

**Table 4:** Pairwise F_ST_ (95% Confidence Interval) among Brazilian regions and grouped according to color.

	Regions				Color	
	North	Northeast	Southeast		White	Brown
Northeast	0.0008 (0–0.0061)			Brown	0.0030 (0–0.0076)	
Southeast	0.0024 (0–0.0086)	0.0031 (0–0.0108)		Black	0.0099 (0.0049–0.0173)	0.0013 (0–0.0063)
South	0.0015 (0–0.0082)	0.0033 (0–0.0117)	0 (0–0.0028)			

Table 5 shows the frequencies of alleles classified by predicted activity. Alleles for reduced function (CYP2D6*9, *10, *17, *29, *41) were observed in 18% of the chromosomes investigated, 15.1% were non-functional alleles (CYP2D6*3, *4, *4×2, *5), 64% were functional alleles (CYP2D6*1, *2A, *35, *39), and 2.6% resulted in increased enzyme activity (CYP2D6*1×2, *1×3, *2×2, *35×2, *2×5). No differences in the prevalence of alleles grouped by function were observed among Brazilian regions (χ^2^ = 10.7; degrees of freedom = 9; p-value = 0.29).

**Table 5:** *CYP2D6* allele predicted activity frequencies (%) in Brazilians.

Predicted activity^a^	Alleles^a^	Total sample	North	Northeast	Southeast	South
None	*3, *4, *4×2, *5	15.1	14.7	15.9	14.3	15.4
Reduced	*9, *10, *17, *29, *41	18.0	14.2	17.8	21.4	18.4
Normal	*1, *2, *35, *39	64.3	68.2	63.2	61.9	64.2
High^b^	*1×2, *1×3, *2×2, *35×2, *2×5	2.6	2.9	3.1	2.4	2.0
Total number of chromosomes		2011	484	517	509	501

a Not all alleles have been functionality determined.

b Duplications in heterozygous individuals that could not be unambiguously assigned to an allele were not included.

χ^2^ = 10.7; degrees of freedom = 9; p-value = 0.29.

Predicted phenotypes (UM, EM, IM, PM) frequencies stratified by Brazilian region and self-reported color are shown in Table 6. As expected, the most frequent predicted metabolic status was EM (83.5%). Overall 2.5% and 3.7% of Brazilians were PMs and UMs respectively. No differences were observed in predicted phenotype frequencies among Brazilian regions (p = 0.467) or self-reported skin color (p = 0.089).

**Table 6:** CYP2D6 predicted phenotype frequencies according to self-reported color and geographical region.

	North			Northeast			Southeast			South		
Phenotype	White	Brown	Black	White	Brown	Black	White	Brown	Black	White	Brown	Black
PM	1.4	3.4	4.7	3.4	3.4	-	1.2	4.6	1.2	4.6	1.2	1.2
IM	4.1	-	10.6	3.4	4.5	8.2	6.9	7.0	12.9	5.7	12.7	8.6
EM	85.1	88.6	80.0	86.4	86.4	83.5	82.7	81.5	77.7	87.4	82.7	79.0
UM	6.7	3.4	4.7	3.4	2.3	5.9	4.6	4.6	1.2	2.3	-	6.2
ND^a^	2.7	4.6	-	3.4	3.4	2.4	4.6	2.3	7.0	-	3.4	5.0
Total number of individuals	74	87	85	88	88	85	87	86	85	87	87	81

a Not Determined.

The Multinomial Log-Linear analysis: self-reported skin color is not associated with the predicted phenotype (p = 0.089); region is not associated with the predicted phenotype (p = 0.467).

Genomic ancestry based on the individual proportions of European, African and Amerindian ancestry independent of self-reported color was investigated in this cohort as a continuous variable. African (p<0.001) and European (p<0.01) proportions were significantly different among predicted phenotypes whereas Amerindian proportions did not vary among these categories (Table 7). The IM predicted phenotype is associated with a higher proportion of African ancestry and a lower proportion of European ancestry in Brazilians.

**Table 7:** Genetic ancestry (mean ± standard deviation) proportions according to CYP2D6 phenotypes.

CYP2D6 phenotype	Amerindian	African	European
PM	0.145±0.164	0.168±0.222	0.687±0.257
IM	0.099±0.098	0.387^b^±0.308	0.514^c^±0.318
EM	0.117±0.146	0.247±0.265	0.636±0.304
UM	0.088±0.118	0.242±0.269	0.670±0.298
p^a^	0.314	0.001	0.013

a P-value for the Kruskal-Wallis One-Way ANOVA.

b Significantly higher mean in the pairwise comparisons. FDR adjusted p-values for the IM×PM, IM×EM, and IM×UM comparisons are, respectively: 0.0014, 0.011, and 0.048.

c Significantly higher mean in the pairwise comparisons with EM and UM phenotypes. FDR adjusted p-values for the IM×EM, and IM×UM comparisons are, respectively: 0.018, 0.027.

## Discussion

This is the most comprehensive study of variation at the *CYP2D6* locus in the Brazilian population. A total of 18 different *CYP2D6* alleles including duplications were identified in this Brazilian cohort, displaying a unique and complex allele distribution. In a country of continental dimensions like Brazil with distinct immigration patterns and different admixture processes, a homogeneous distribution of *CYP2D6* alleles across geographical regions and skin color categories was unexpected. The homogeneity seen is in contrast with the results of previous pharmacogenetic studies in this population that demonstrated a highly heterogeneous distribution of several pharmacogene polymorphisms across geographical regions, as well as within the color/race categories adopted by the Brazilian census [21]–[27].

There are marked differences in *CYP2D6* allele frequencies in populations of different continental origins. Certain alleles were observed in high frequencies in different populations such as CYP2D6*4 in Europeans, CYP2D6*10 in Asians and CYP2D6*17 in Africans [5],[28]. In Brazilians the prevalence of CYP2D6*4 (9.2%) and CYP2D6*17 (5.6%) are intermediate between those described for Europeans and Africans as expected for an admixed population.

Few *CYP2D6* screening surveys were previously performed in Brazilian subjects. Two studies described CYP2D6*3 and CYP2D6*4 allele frequencies in a southeastern population based on one SNP PCR-RFLP methodology. These allele frequencies were 0.04 and 0.14 for white subjects and 0.03 and 0.10 for black individuals, respectively, which are higher than those described herein [29],[30]. Larger studies were performed in the southern Brazilian population [31]–[33]. Overall the allele frequencies were very similar to those observed herein, except for CYP2D6*4 which was observed in 18% of breast cancer patients [33]. These discrepancies in results among studies possibly reflect methodological issues such as sample sizes, subject characteristics such as different admixture proportions, healthy subjects or disease patients or different study designs.

The frequency of *CYP2D6* duplications/multiplications alleles carriers is about 1–2% in Northern Europe [12], increases to 7–10% in the Mediterranean region [34],[35], and become as high as 29% in Ethiopia [36], but in Sub-Saharan Africans the median of duplications/multiplications alleles is about 7% only [12]. In the present study, 87 of the 1020 volunteers (8.5%) were found to carry a duplication/multiplication allele, but only 2.6% of those alleles could be unambiguously assigned as functional alleles (CYP2D6*1×2, *1×3, *2×2, *35×2, *2×5).

In Brazil as in all continental groups, EM was the most common phenotype. Native South American individuals belong to either the UM or the EM class which predicts high metabolic capacity of these populations [12]. The prevalence of the PM phenotype appears to be higher among Europeans, whereas higher heterogeneity has been reported in the African continent where the prevalence of PMs ranged from 0 to 19% [2],[9],[12]. This phenotype is less frequent in Asian populations [12],[37]. The frequency of predicted PMs in Brazilians was 2.5% independent of region or ancestry, which is similar to the prevalence of PMs reported in North American Latino populations (2.2%–6.6%) [9].

In Brazil, the second most common predicted phenotype was IM (7.1%). This finding is due, at least in part, to CYP2D6*17 (5.6%), CYP2D6*29 (3.4%), and CYP2D6*41 (5.5%) alleles. Common decreased-function variants, CYP2D6*10, *17 and *41, led to higher number of IMs in East Asia, Africa and Middle East respectively [2],[12]. In the admixed Brazilian population investigated herein this phenotypic class was associated with higher African and consequently with lower European ancestries proportions. Although no differences by race/color were observed among CYP2D6 phenotype frequencies we demonstrated that the influence of ancestry in Brazilians is better explained by the individual proportions of African and European ancestry as a continuous variable as previously shown in other pharmacogenetic studies in the Brazilian population [26],[27],[38]. However, this influence is restricted to the IM predicted phenotype.

Typical substrates for CYP2D6 are largely lipophilic bases and include some antidepressants, antipsychotics, antiarrhythmics, antiemetics, beta-blockers and opioids. In the Brazilian population, the frequency of PMs owing to *CYP2D6* polymorphisms is 2.5% and 3.7% are UMs. Both groups have an altered capacity to metabolize some drugs and could thus potentially benefit from genotyping for *CYP2D6* when treated with any of these drugs. Subjects with multiple gene copies will metabolize drugs more rapidly and therapeutic plasma levels will not be achieved at ordinary drug dosages, whereas individuals lacking functional *CYP2D6* alleles metabolize CYP2D6 substrates at a lower rate, and the risk for adverse drug reactions is higher [39],[40]. The CYP2D6 IMs constitute 7%, but these patients’ potential to benefit from genotyping for *CYP2D6* is less clear. The importance of the IM phenotype is further complicated by drug specificity. CYP2D6*17, which is frequent in Brazilians, was described as being associated with reduced activity for several CYP2D6 substrates. However, CYP2D6*17 appears to be associated with normal activity for risperidone [41].

This work should be interpreted in the context of some limitations. First, we did not screen all described SNPs, but we investigated all alleles with frequencies higher than 1% in the Americas [42], except CYP2D6*12 and CYP2D6*82 but these alleles were found only in native Argentinian-Paraguayan and Mexican Amerindians respectively. Therefore, some alleles such as CYP2D6*6 were not observed and/or its tag SNP was not included because its global frequency is less than 1% including previous Brazilian samples [12],[31]–[33], as well as the CYP2D6*13-like *CYP2D7/2D6* hybrid genes, which are fairly rare (0.1–0.2%) in European and African populations [42]. Second, some alleles could not be unambiguously detected because their SNP combination did not match any known *CYPD6* alleles (Table S2). Although we ruled out genotyping errors employing new independent genotyping of these samples, we did not resequence them to eventually describe new variants because this procedure was beyond the scope of this exploratory study. Third, we estimated copy number based on only one assay that targets exon 9. Although some authors claim that this strategy has limitations and pitfalls [43],[44], these limitations seem to be restricted to Asians where exon 9 conversions appear at higher frequencies. However, in other ethnic groups, one study compared four *CYP2D6* regions (exon 1, intron 5, intron 6, and exon 9) in copy number determination and the four regions resulted in robust copy number assignments that were in agreement with genotype, sequencing and extra-long PCR-based data [44]. Another investigation described that the TaqMan assay targeting exon 9 revealed high sensitivity and specificity [45]. Recently Fang *et al.* [46] compared all the three TaqMan real-time PCR assays for copy number determination (intron 2, intron 6, exon 9) and they showed high concordant results. The CYP2D6*5 allele was also confirmed by long-range PCR.

Implementing pharmacogenetics in clinical practice has proven to be a challenge worldwide, and it is expected that this would be an even greater challenge in admixed populations because of their heterogeneity and genetic diversity. The Clinical Pharmacogenetics Implementation Consortium (CPIC) Guideline for codeine and tricyclic antidepressants therapies [28],[47],[48] strongly recommends *CYP2D6* testing before codeine use, for other drugs the potential genotyping benefits are less clear. PM and UM classes did not vary among regions and/or ancestry proportions therefore unique guidelines for Brazilians are possible and could potentially avoid ineffective or adverse events outcomes.

## Materials and Methods

### Ethics Statement

The Ethics Committees of the Instituto Nacional de Cancer (INCA), Rio de Janeiro, Universidade Federal de Minas Gerais, Universidade Federal do Rio Grande do Sul, Universidade Federal do Ceará, and Universidade Federal do Pará approved the study as well as the written Informed Consent form. The samples were anonymized after collection. Each individual signed a written informed consent.

### Subjects

The study cohort consisted of 1020 healthy adults recruited from the north, northeast, south and southeast regions of Brazil. Sample collection and the individual ancestry determination were fully described previously [24]. Briefly, each individual was asked to self-identify according to the classification scheme adopted by the official Brazilian Census [49], which relies on self-perception of skin color. The subjects were distributed into the following three groups: white (n = 336), brown (n = 349), and black (n = 335).

### Laboratory procedures

Genomic DNA was isolated from peripheral blood by standard procedures. *CYP2D6* SNPs (Table 8; Table S3) were determined by allelic discrimination with TaqMan SNP Genotyping Assays according to the manufacturer’s recommended protocols. *CYP2D6* gene deletion and/or duplications were identified by a TaqMan Copy Number Assay. Hs00010001_cn specifically targets *CYP2D6* exon 9 sequences and will not amplify *CYP2D7* or *CYP2D8* pseudogenes, or *CYP2D6* alleles having *CYP2D7* sequences in exon 9 (e.g. CYP2D6*36). An assay for Ribonuclease P RNA component H1 gene (*RNase P,* assay ID 4403326) was used as reference to determine copy number. The duplex-PCR reaction was performed according to the manufacturer’s instructions. Samples carrying one and three copies were included in all plates as *CYP2D6* copy number controls. The amplification products were analyzed with the CopyCaller software v2.0. Reactions were considered acceptable if confidence >95% and Z score <1.75.

**Table 8:** *CYP2D6* genotyped polymorphisms, inferred alleles and estimated enzyme activity.

CYP2D6 allele	Nucleotide Position in the CYP2D6 gene^a^											CYP2D6 duplication	Enzyme activity^a^
	−1584	31	100	1023	1846	2549	2615	2850	2988	3183	4180		
*1	C	G	C	C	G	A	A	C	G	G	G	−	Normal
*2	**G**	G	C	C	G	A	A	**T**	G	G	**C**	−	Normal
*3	C	G	C	C	G	**Del**	A	C	G	G	G	−	None
*4	C	G	**T**	C	**A**	A	A	C	G	G	**C**	−	None
*5^b^	−	−	−	−	−	−	−	−	−	−	−	−	None
*9	C	G	C	C	G	A	**Del**	C	G	G	G	−	Decr
*10	C	G	**T**	C	G	A	A	C	G	G	**C**	−	Decr
*17	C	G	C	**T**	G	A	A	**T**	G	G	**C**	−	Decr
*29	C	G	C	C	G	A	A	**T**	G	**A**	**C**	−	Decr
*34	C	G	C	C	G	A	A	**T**	G	G	G	−	ND
*35	**G**	**A**	C	C	G	A	A	**T**	G	G	**C**	−	Normal
*39	C	G	C	C	G	A	A	C	G	G	**C**	−	Normal
*41	C	G	C	C	G	A	A	**T**	**A**	G	**C**	−	Decr
*1x	C	G	C	C	G	A	A	C	G	G	G	+	Incr
*2x	**G/C**	G	C	C	G	A	A	**T**	G	G	**C**	+	Incr
*4x	C	G	**T**	C	**A**	A	A	C	G	G	**C**	+	None
*17x	C	G	C	**T**	G	A	A	**T**	G	G	**C**	+	ND
*3x	**G**	**A**	C	C	G	A	A	**T**	G	G	**C**	+	Incr

Nucleotides in bold are the polymorphic sites.

Decr = decreased activity.

Incr = increased activity.

ND = activity not determined.

a According to The Human Cytochrome P450 (CYP) Allele Nomenclature Database [33].

b Deletion of the entire *CYP2D6* gene.

SNPs ID numbers are listed in Table S2.

### Definition of alleles, genotypes and phenotype classes

Alleles were inferred using the software PHASE v.2.1. As parameters 100 burn-in steps followed by 10,000 Markov Chain Monte Carlo iterations were used [50],[51]. The haplotypes generated by Phase were compared to the *CYP2D6* allele nomenclature of The Human Cytochrome P450 (CYP) Allele Nomenclature Database [8]. For allele designation only perfect matches were considered. The CYP2D6*1 allele was set when no nucleotide change was observed in all genotyped SNPs (Table 8). When Phase software inferred a SNP combination that could not be assigned to a known allele, they were pooled as others (Table S3 shows more details about these alleles).

The prediction of enzyme activity corresponding to each haplotype was based on The Human Cytochrome P450 (*CYP*) Allele Nomenclature Database [8]. This classification of phenotypes is based on the assumption of dominance, in which the phenotype is determined by the most efficient haplotype in the genotype. Based on these assumptions metabolic status/genotype (predicted phenotype) was defined as: ultrarapid metabolizer (UM): at least three active gene copies; extensive metabolizer (EM): one or two active alleles; intermediate metabolizer (IM): two reduced activity alleles or one reduced activity and one inactive allele; poor metabolizer (PM): two inactive alleles [13],[41]. Other IM classifications could also be used such as the CPICs guidelines. But, these guidelines are gene/drug guidelines. They used a system that assigned score values to the allele activity. The classification of patients with an activity score of 1.0 (two reduced function alleles or one functional and one nonfunctional allele) as EM in the codeine CPIC was based on specific data for morphine to codeine formation in these patients. In the tricyclic antidepressants CPIC [48], they used the same classification because pharmacokinetics studies suggested that patients with an activity score of 1.0 have a higher CYP2D6 metabolic capacity for these drugs as compared with patients with an activity score of 0.5 (that would be the one reduced function and one nonfunctional allele, the IM phenotype for them). As our study is not drug/substrate specific, we decided to use the IM and EM phenotype designations widely used in the literature [9],[13],[14],[31],[32],[41].

### Statistical analyses

Allele and genotype frequencies were estimated by gene counting. Genetic diversity based on allele frequencies was assessed by analysis of molecular variance (AMOVA) [52]–[54]. Two levels of diversity were assessed: among regions and among color/race strata. Inter-region variability was determined by F_ST_ and their 95% confidence intervals were estimated with diveRsity package [55] in the R language and environment [56]. The parameter used was 3,000 bootstraps.

Chi-square test was performed to compare the *CYP2D6* allele and phenotypes among regions. Statistical associations between predicted phenotype and self-reported skin color or geographical region were inferred by fitting multinomial log-linear models. This procedure obviates the need for correction for multiple comparisons because the main effects and interaction terms are tested simultaneously within each regression context. Kruskal–Wallis test was performed to examine the association between the predicted phenotype and the genetic ancestry, followed by the Mann-Whitney U test for pairwise comparisons. The pairwise p-values were adjusted for the False Discovery Rate (FDR) [57]. Statistical analysis was performed using the SPSS18.0 statistical package for Windows), WinPEPI [58], and the Arlequin Software v.3.0 [59]. P-value<0.05 was considered significant in all analyses.

## Supporting Information

Table S1 ***CYP2D6*** **Genotypes observed in Brazilians.**
(DOCX)

Table S2 **Description of the alleles called Others.**
(DOC)

Table S3 **Variants of the** ***CYP2D6*** **genotyped and their SNP ID number.**
(DOC)
