# Supplementary material for: Distribution of CYP2D6 Alleles and Phenotypes in the Brazilian Population
Source: PLoS One. 2014 Oct 20;9(10):e110691. doi: 10.1371/journal.pone.0110691 (PMC4203818; doi:10.1371/journal.pone.0110691)
Supplement: Table S1 — CYP2D6 Genotypes observed in Brazilians. (DOCX) [file pone.0110691.s001.docx]

Table S1: *CYP2D6* Genotypes observed in Brazilians

| Genotype | Copy Number | Number of individuals carrying the genotype |
| --- | --- | --- |
| Predicted phenotype: EM | |  |
| **1/*1* | 2 | 168 |
| **1/*2* | 2 | 143 |
| **1/*4* | 2 | 67 |
| **1/*17* | 2 | 50 |
| **2/*2* | 2 | 50 |
| **2/*4* | 2 | 45 |
| **1/*41* | 2 | 44 |
| **1/*5* | 1 | 43 |
| **1/*29* | 2 | 25 |
| **1/*35* | 2 | 24 |
| **2/*41* | 2 | 24 |
| **2/*17* | 2 | 22 |
| **2/*5* | 1 | 20 |
| **2/*10* | 2 | 11 |
| **1/*9* | 2 | 10 |
| **1/*3* | 2 | 9 |
| **2/*29* | 2 | 9 |
| **2/*35* | 2 | 8 |
| **1/*10* | 2 | 6 |
| **4/*35* | 2 | 6 |
| **1/*4^a^* | 3 | 6 |
| **1/*39* | 2 | 5 |
| **35/*41* | 2 | 5 |
| **1/other* | 2 | 5 |
| **2/*4^a^* | 3 | 5 |
| **2/*9* | 2 | 4 |
| **39/*39* | 2 | 4 |
| **1x2/*29* | 3 | 3 |
| **2/*3* | 2 | 3 |
| **2/*41^a^* | 3 | 3 |
| **2/*35* | 2 | 3 |
| **1/*41^a^* | 3 | 3 |
| **1/*17^a^* | 3 | 2 |
| **2/*10^a^* | 3 | 2 |
| **2/*17^a^* | 3 | 2 |
| **10/*35* | 2 | 2 |
| **1/*10^a^* | 3 | 1 |
| **1/*17* | ND | 1 |
| **1/*34* | 2 | 1 |
| **2x2/*29* | 3 | 1 |
| **4/*35^a^* | 3 | 1 |
| **4/*39* | 2 | 1 |
| **5/*35* | 1 | 1 |
| **35/*39* | 2 | 1 |
| **35/*41^a^* | 3 | 1 |
| **39/*other* | 2 | 1 |
| Predicted phenotype: UM | |  |
| **1/*2^a^* | 3 | 17 |
| **1/*1x2* | 3 | 6 |
| **2/*2x2* | 3 | 4 |
| **1/*2^a^* | 4 | 3 |
| **2x2/*2x2* | 4 | 2 |
| **1x2/*1x2* | 4 | 1 |
| **1x2/*1x3* | 5 | 1 |
| **1/*2^a^* | 5 | 1 |
| **2/*35^a^* | 3 | 1 |
| **2x5/*other* | 6 | 1 |
| **35x2/*39* | 3 | 1 |
| Predicted phenotype: PM | |  |
| **4/*5* | 1 | 10 |
| **4/*4* | 2 | 8 |
| **5/*5* | 0 | 4 |
| **4/*4x2* | 3 | 2 |
| **4x2/*4x2* | 4 | 1 |
| **3/*5* | 1 | 1 |
| Predicted phenotype: IM | |  |
| **4/*41* | 2 | 14 |
| **4/*29* | 2 | 6 |
| **5/*29* | 1 | 5 |
| **10/*17* | 2 | 5 |
| **10/*41* | 2 | 4 |
| **17/*29* | 2 | 4 |
| **5/*41* | 1 | 3 |
| **9/*29* | 2 | 3 |
| **10/*29* | 2 | 3 |
| **29/*41* | 2 | 3 |
| **4/*17* | 2 | 2 |
| **5/*10* | 1 | 2 |
| **29/*29* | 2 | 2 |
| **41/*41* | 2 | 2 |
| **3/*41* | 2 | 1 |
| **4/*9* | 2 | 1 |
| **4/*9^a^* | 3 | 1 |
| **4/*10* | 2 | 1 |
| **4/*10^a^* | 4 | 1 |
| **4/*17^a^* | 3 | 1 |
| **5/*9* | 1 | 1 |
| **9/*10* | 2 | 1 |
| **9/*17* | 2 | 1 |
| **10/*10* | 2 | 1 |
| **10/*17^a^* | 3 | 1 |
| **17/*17* | 2 | 1 |
| **17/*41* | 2 | 1 |
| **17x2/*29* | 3 | 1 |

ND = copy number was not determined

^a^ The duplication could not be unambiguously assigned to one allele, but the phenotype could be predicted because in those cases it is independent of which allele is duplicated
