# Supplementary material for: Distribution of CYP2D6 Alleles and Phenotypes in the Brazilian Population
Source: PLoS One. 2014 Oct 20;9(10):e110691. doi: 10.1371/journal.pone.0110691 (PMC4203818; doi:10.1371/journal.pone.0110691)
Supplement: Table S2 — Description of the alleles called Others. (DOC) [file pone.0110691.s002.doc]

Table S2: Description of the alleles called Others

| SNPs present | Possible allele in which the tag SNP was not genotyped | Number of individuals |
| --- | --- | --- |
| -1584G | *CYP2D6*71* | 3 |
| -1584G | *CYP2D6*71* | 1 |
| -1584G | *CYP2D6*71* | 1 |
| -1584G, 2850T | *CYP2D6*63* | 2 |
| -1584G, 2850T | *CYP2D6*63* | 1 |
| -1584G, 2850T | *CYP2D6*63* | 1 |
| -1584G, 2850T, 2988A, 4180C | None | 1 |
| 100T | *CYP2D6*68* | 1 |
| 100T, 1023T, 1849A, 4180C | None | 14 |
| 2850T, 3183A | None | 2 |
