# Supplementary material for: Distribution of CYP2D6 Alleles and Phenotypes in the Brazilian Population
Source: PLoS One. 2014 Oct 20;9(10):e110691. doi: 10.1371/journal.pone.0110691 (PMC4203818; doi:10.1371/journal.pone.0110691)
Supplement: Table S3 — Variants of the CYP2D6 genotyped and their SNP ID number. (DOC) [file pone.0110691.s003.doc]

Table S3: Variants of the *CYP2D6* genotyped and their SNP ID number

| Variant | SNP ID number |
| --- | --- |
| -1584C>G | rs1080985 |
| 31G>A | rs769258 |
| 100C>T | rs1065852 |
| 1023C>T | rs28371706 |
| 1846G>A | rs3892097 |
| 2549A>del | rs35742686 |
| 2615_2617delAAG | rs28371720 |
| 2850C>T | rs16947 |
| 2988G>A | rs28371725 |
| 3183G>A | rs59421388 |
| 4180G>C | rs1135840 |
